# Acute kidney injury in SARS-CoV2-related pneumonia ICU patients: a retrospective multicenter study

**DOI:** 10.1186/s13613-021-00875-9

**Published:** 2021-05-31

**Authors:** Guillaume Geri, Michael Darmon, Lara Zafrani, Muriel Fartoukh, Guillaume Voiriot, Julien Le Marec, Saafa Nemlaghi, Antoine Vieillard-Baron, Elie Azoulay

**Affiliations:** 1 Medical Intensive Care Unit, Ambroise Paré Hospital, AP-HP, 9 avenue Charles de Gaulle, 92100 Boulogne-Billancourt, France; 2 Paris Saclay University, Gif-sur-Yvette, France; 3 INSERM UMR 1018, CESP, Villejuif, France; 4 FHU SEPSIS, Saclay, France; 5 Medical Intensive Care Unit, Saint Louis Hospital, AP-HP, Paris, France; 6 Paris University, Paris, France; 7 INSERM U1153, Centre of Research in Epidemiology and Statistics, Paris, France; 8 INSERM U976, Immunologie Humaine, Pathophysiologie et immunothérapie, Paris, France; 9 Medical Intensive Care Unit, Tenon Hospital, AP-HP, Paris, France; 10 Paris Sorbonne University, Paris, France; 11 INSERM U955 (IMRB), Equipe GEIC2O, 94000 Créteil, France; 12 Medical Intensive Care Unit, Pitié-Salpétrière Hospital, AP-HP, Paris, France; 13 INSERM, UMRS1158 Neurophysiologie Respiratoire Expérimentale et Clinique, Paris, France

**Keywords:** Acute kidney injury, COVID-19, Renal replacement therapy

## Abstract

**Background:**

While acute kidney injury (AKI) is frequent in severe SARS-CoV2-related pneumonia ICU patients, few data are still available about its risk factors.

**Methods:**

Retrospective observational study performed in four university affiliated hospitals in Paris. AKI was defined according to the KIDGO guidelines. Factors associated with AKI were picked up using multivariable mixed-effects logistic regression. Independent risk factors of day 28 mortality were assessed using Cox model.

**Results:**

379 patients (median age 62 [53,69], 77% of male) were included. Half of the patients had AKI (*n* = 195, 52%) including 58 patients (15%) with AKI stage 1, 44 patients (12%) with AKI stage 2, and 93 patients (25% with AKI stage 3). Chronic kidney disease (OR 7.41; 95% CI 2.98–18.4), need for invasive mechanical ventilation at day 1 (OR 4.83; 95% CI 2.26–10.3), need for vasopressors at day 1 (OR 2.1; 95% CI 1.05–4.21) were associated with increased risk of AKI. Day 28 mortality in the cohort was 26.4% and was higher in patients with AKI (37.4 vs. 14.7%, *P* < 0.001). Neither AKI (HR 1.35; 95% CI 0.78–2.32) nor AKI stage were associated with mortality (HR [95% CI] for stage 1, 2 and 3 when compared to no AKI of, respectively, 1.02 [0.49–2.10], 1.73 [0.81–3.68] and 1.42 [0.78–2.58]).

**Conclusion:**

In this large cohort of SARS-CoV2-related pneumonia patients admitted to the ICU, AKI was frequent, mostly driven by preexisting chronic kidney disease and life sustaining therapies, with unclear adjusted relationship with day 28 outcome.

**Supplementary Information:**

The online version contains supplementary material available at 10.1186/s13613-021-00875-9.

## Background

The outbreak of severe acute respiratory syndrome (SARS-CoV2)-related disease has widely spread for almost a year [1]. The clinical presentation is mainly respiratory, potentially leading to severe respiratory failure [2]. Such a respiratory failure is complex and related to at least several factors including viral direct lung injury as well as multiple microthrombosis [3].

Kidney involvement is common in COVID-19 patients and may be observed in up to 40% of cases [4]. Different histological phenotypes from acute tubular necrosis up to specific glomerular involvement as collapsing glomerulopathy [5–7] have been reported. Pathogenesis of AKI during COVID-19 remains imperfectly understood. Besides cytokine storm, kidney congestion related to right ventricular failure or interactions with mechanical ventilation, endothelitis may be the cornerstone of organ—and especially the kidney—involvement in SARS-CoV2-related pneumonia.

However, few data are still available about risk factors associated with AKI occurrence and the relationship between inflammatory biomarkers and renal failure.

In the present study, we aimed to evaluate factors associated with AKI occurrence as well as its impact on day 28 mortality in SARS-CoV2 patients admitted to the ICU in four intensive care units from the Parisian area.

### Patients and methods

#### Study design, setting and participants

This retrospective observational study was performed in four university-affiliated hospitals in Paris, as previously reported [8]. Consecutive patients with laboratory-confirmed SARS-CoV-2 infection admitted to one of the ICUs between 21 February and 24 April 2020 were enrolled. The appropriate ethics committee approved the study and waived the need for informed consent in accordance with French legislation about retrospective studies. Laboratory confirmation of SARS-CoV-2 was defined as a positive real-time reverse transcriptase–polymerase chain reaction (RT–PCR) assay of nasal and pharyngeal swabs [9].

### Data collection

Data were recorded by the intensivists in each ICU. The variables reported in the tables and figures were abstracted from the medical charts and electronic reports. Causes of immunosuppression included solid tumours, haematological malignancies, solid organ transplantation, long-term immunosuppressive therapy (i.e., high-dose steroids (> 1 mg/kg whatever the duration) or any immunosuppressant for more than 3 months), and HIV infection [10]. Obesity was defined as previously reported [11]. The SOFA score was calculated within 24 h of ICU admission [12]. Regards to renal data, day 1 serum creatinine was collected as well as urine output within the first 24 h. To note that some of the patients included from the Saint Louis hospital were already reported elsewhere [13].

### Definition of AKI

AKI was defined according to both urinary output and serum creatinine KDIGO criteria {KidneyDiseaseImprovingGlobalOutcomesKDIGOAcuteKidneyInjuryWorkGroup:2012gn} as follows: stage 1—increase in serum creatinine by 0.3 mg/dl within 48 h or a 1.5 to 1.9 times increase in serum creatinine from baseline or urinary output < 0.5 ml/kg/h for 6–12 h within 7 days; stage 2–2.9 times increase in serum creatinine or urinary output < 0.5 ml/kg/h for ≥ 12 h within 7 days; stage 3–3 times or more increase in serum creatinine or to ≥ 4.0 mg/dl or initiation of RRT or urinary output < 0.3 ml/kg/h for ≥ 24 h or anuria for ≥ 12 h within 7 days. Patients were stratified according to the highest AKI stage attained during the first 7 days of ICU stay.

Baseline creatinine was defined as the best value in the 3 preceding months or if unavailable as the lowest value during ICU stay or was back calculated based on a glomerular filtration rate of 60 mL/min/1.73m2 with MDRD equation in patients without known chronic kidney disease. Chronic kidney disease (CKD) was defined according to the KDIGO definition.

### Statistical analysis

Descriptive statistics were provided as *n*(%) and median [interquartile] or mean (standard deviation) for categorical and continuous variables, respectively. Comparisons were performed across KDIGO stages of AKI using Pearson Chi-square test and Kruskal–Wallis or ANOVA for categorical and continuous variables, as appropriate.

No imputation for missing data was performed in this analysis and rate of missing data in the overall data set used for this study was 6.8%.

Factors associated with AKI risk were assessed using conditional stepwise regression with 0.2 as the critical *P* value for entry into the model, and 0.1 as the *P* value for removal. Interactions and correlations between the explanatory variables were carefully checked. Continuous variables for which log linearity was not confirmed were transformed into categorical variables according to median or IQR. The final models were assessed by calibration, discrimination and relevancy. Residuals were plotted, and the distributions inspected. The final model was planned to be a mixed model taking center effect as random effect on the intercept into account. It was planned to force one by one oxygenation modality, PaO_2_/FiO_2_ ratio, procalcitonin and fibrinogen at ICU admission, in the final model should these variables not be selected.

Independent risk factors of day 28 mortality were assessed using Cox model. Conditional stepwise variable selection was performed with 0.2 as the critical *P* value for entry into the model, and 0.1 as the *P* value for removal. It was planned, should this variable not be selected, to force one by one, AKI and AKI severity should these variables not be selected. Interactions and correlations between the explanatory variables were carefully checked. Validity of proportional hazards assumption, influence of outliers, and linearity in relationship between the log hazard and the covariates were carefully checked. Final model was the selected model including frailty effect on center.

Survival curve were plotted using Kaplan Meier curves and influence of AKI on outcome was compared using log-rank test.

All tests were two-sided, and *P* values less than 0.05 were considered statistically significant. Analyses were done using R software version 3.6.2 (https://www.r-project.org), including survival, lme4, lmerTest, givitiR packages.

## Results

### Baseline characteristics

Overall, 379 COVID-19 patients were admitted in the participating ICUs during the study periods (Additional file 1: Figure S1). Main patients’ characteristics are described in Table 1. Median age was 62 years [interquartile 53,69] and 291 patients (77%) were of male gender. Hypertension (*n* = 188, 50%), diabetes (*n* = 114, 30%), underlying immune defect (*n* = 68, 18%) and chronic kidney disease (*n* = 65, 17%) were the main comorbidities. Two hundred and forty-seven patients were obese (*n* = 137, 39%) or overweight (*n* = 110, 31%). Median SOFA score at admission was 5 [3–8]. At ICU admission, half of the patients (*n* = 204, 54%) required invasive mechanical ventilation, and 165 (44%) vasopressors. Half of the patients had AKI (*n* = 195, 52%) including 58 patients (15%) with AKI stage 1, 44 patients (12%) with AKI stage 2, and 93 patients (25% with AKI stage 3).

**Table 1:** Patients characteristics according to occurrence of AKI

	No AKI patients	AKI patients	*P* value
*n*	184	**195**	
Age (years)	60 [52–68]	63 [54–69]	0.03
Male gender	129 (70.1%)	162 (83.1%)	0.004
Past medical history			
Chronic obstructive pulmonary disease	5 (2.7%)	15 (7.7%)	0.05
Asthma	13 (7.1%)	10 (5.1%)	0.57
Hypertension	72 (39.1%)	116 (59.5%)	< 0.001
Diabetes	44 (23.9%)	70 (35.9%)	0.02
Immune defect	27 (14.7%)	41 (21.2%)	0.13
Chronic heart failure	8 (4.3%)	24 (12.3%)	0.009
Chronic kidney disease	12 (6.5%)	53 (27.2%)	< 0.001
Body mass index, kg/m^2^	29 [26–36]	29 [26–32]	0.14
Body weight class			0.63
Overweight	48 (28.9%)	62 (32.6%)	
Obese	68 (41.0%)	69 (36.3%)	
Other	50 (30.1%)	59 (31.1%)	
NSAIDs use before ICU	5 (2.7%)	6 (3.1%)	1.000
Days since onset of disease			0.02
1–7 days	65 (35.5%)	96 (49.7%)	
8–14 days	97 (53.0%)	76 (39.4%)	
> 14 days	21 (11.5%)	21 (10.9%)	
Days between hospital and ICU admission	1 [0–2]	1 [0–3]	0.26
Body Temperature (°C)	37.6 [40.0–38.0]	37.4 [36.3–38.3]	0.63
Digestive tract symptoms	51 (27.7%)	48 (24.6%)	0.57
Laboratory results at ICU admission			
Platelets (G/L)	220 [167–284]	199 [146–263]	0.01
Creatinine (µmol/L)	69 [61–83]	107 [78–173]	< 0.001
C Reactive Protein	159 [100–226]	181 [106–268]	0.05
Procalcitonin (µg/L)	0.3 [0.2–0.6]	0.8 [0.4–1.7]	< 0.001
LDH (UI/L)	537 [454–751]	593 [459–797]	0.19
Fibrinogene (g/L)	7.4 [6.3–7.5]	7.2 [5.8–8.6]	0.10
Ferritin	1157 [687–2060]	1554 [856–2749]	0.09
Ddimeres	1320 [753–2790]	2244 [1025–4003]	0.001
Organ support and dysfunction during first 24 h			
Oxygenation_modality (%)			< 0.001
Standard O2	49 (26.6%)	19 (9.7%)	
HFNO/NIV	74 (40.2%)	33 (16.9%)	
Mechanical_ventilation	61 (33.2%)	143 (73.3%)	
Normal Troponin	136 (80.5%)	70 (40.9%)	< 0.001
SOFA score	3 [2–5]	7 [4–9]	< 0.001
P/F ratio (mmHg)	142 [86–191]	124 [90–195]	0.97
Vasopressors	42 (22.8%)	123 (63.1%)	< 0.001
Need for RRT during ICU stay	10 (5.4%)	64 (32.8%)	< 0.001
Worst KDIGO Stage at day 7			< 0.001
No AKI	184 (100%)	0 (0.0)	
Stage 1	0	58 (29.7%)	
Stage 2	0	44 (22.6%)	
Stage 3	0	93 (47.7%)	
ICU mortality	30 (18.8%)	68 (42.5%)	< 0.001
Hospital Mortality	30 (21.3%)	73 (57.9%)	< 0.001
Day 28 mortality	27 (14.7%)	73 (37.4%)	< 0.001

Qualitative variables are presented as *n*(%) and compared using the Pearson’s Chi-square test or the exact Fisher’s test, as appropriate

Quantitative variables are presented as median [interquartile] and compared using a Mann–Whitney test

### Factors associated with AKI occurrence

**Fig. 1:**
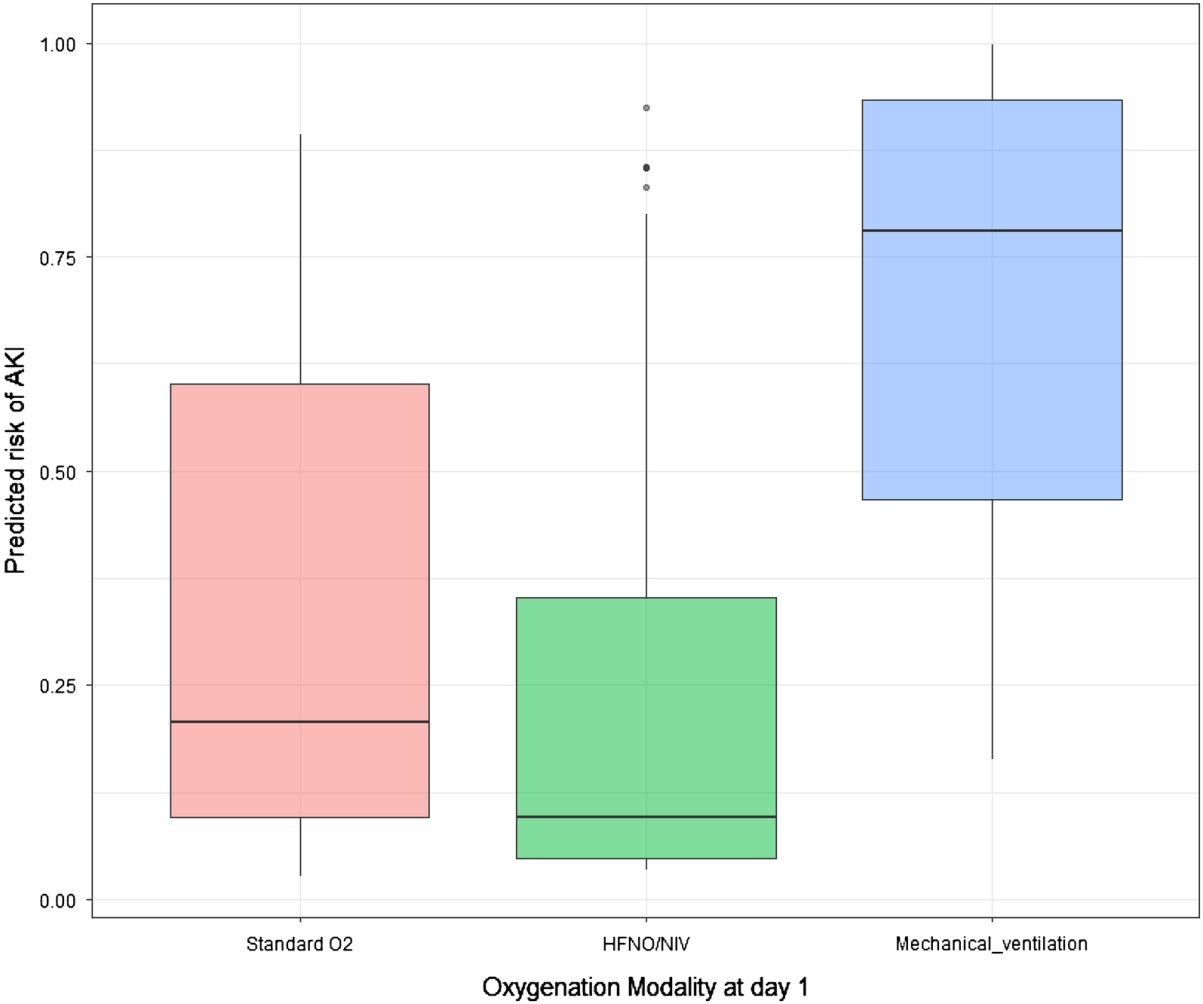
Predicted probability of acute kidney injury obtained using a multivariable model according to oxygen delivery modality

Main factors associated with AKI before adjustment are reported in Table 1. After adjustment for confounders, chronic kidney disease (OR 7.41; 95% CI 2.98–18.4), need for invasive mechanical ventilation at day 1 (OR 4.83; 95% CI 2.26–10.3), need for vasopressors at day 1 (OR 2.1; 95% CI 1.05–4.21) were associated with increased risk of AKI (Table 2). Conversely, normal troponin level at ICU admission (OR 0.49; 95% CI 0.25–0.96) and ICU admission more than 7 days after onset of disease (OR 0.40; 95% CI 0.21–0.76; and OR 0.28; 95% CI 0.09–0.80, respectively, for patients admitted 8–14 days and more than 14 days after onset of disease) were associated with lower risk of AKI (Table 2). Model calibration is reported in Additional file 1: Figure S2. When forced in the final model, neither PaO_2_/FiO_2_ ratio (OR per log10 2.04; 95% CI 0.47–8.76), procalcitonin at ICU admission (OR per µg/L 1.00; 95% CI 0.98–1.02) or fibrinogen level (OR per g/L 1.01; 95% CI 0.99–1.02) were associated with AKI occurrence. Figure 1 reports adjusted risk of AKI according to initial oxygenation modality.

**Table 2:** Mixed logistic regression assessing risk factors of AKI

Variable	OR	95% CI	*P* Value
(Intercept)	0.49	0.11–2.1	0.33
Normal troponin at ICU admission	0.48	0.24–0.96	0.03
Hypertension	1.70	0.92–3.17	0.09
Chronic Kidney Disease	7.40	2.98–18.41	< 0.001
Invasive Mechanical ventilation at day 1	4.83	2.25–10.33	< 0.001
Vasopressors at day 1	2.09	1.05–4.20	0.04
Delay since disease onset < 8 days	Ref	–	NA
Delay since disease onset 8 to 14 days	0.40	0.21–0.76	0.005
Delay since disease onset > 14 days	0.28	0.10–0.80	0.02

Center was entered as random effect on the intercept (model calibration see figure S2; VIF < 1.5 for every of the selected variables)

### Relationship between AKI and ICU mortality

**Fig. 2:**
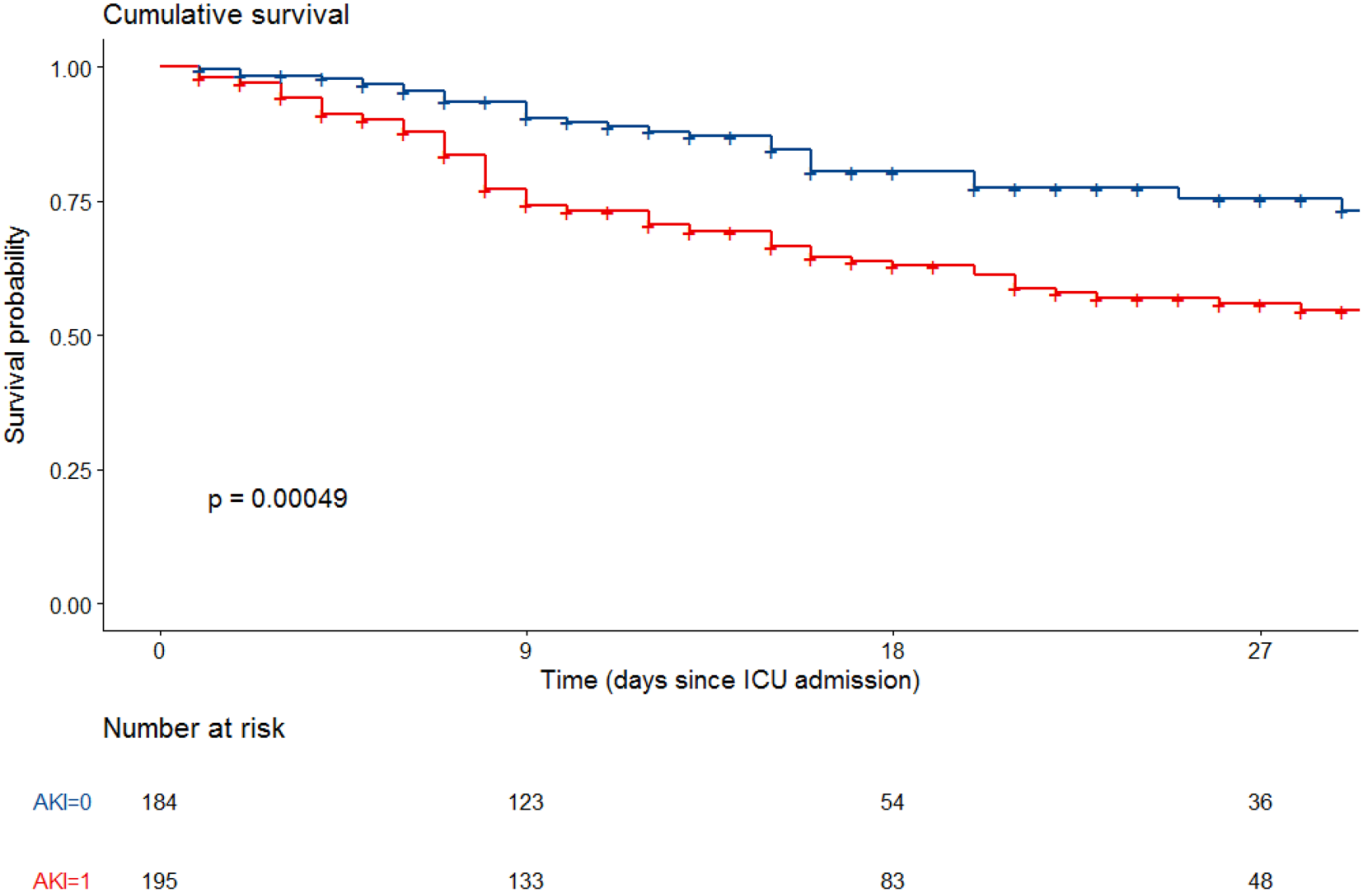
Univariate Kaplan–Meier curves according to acute kidney injury

**Fig. 3:**
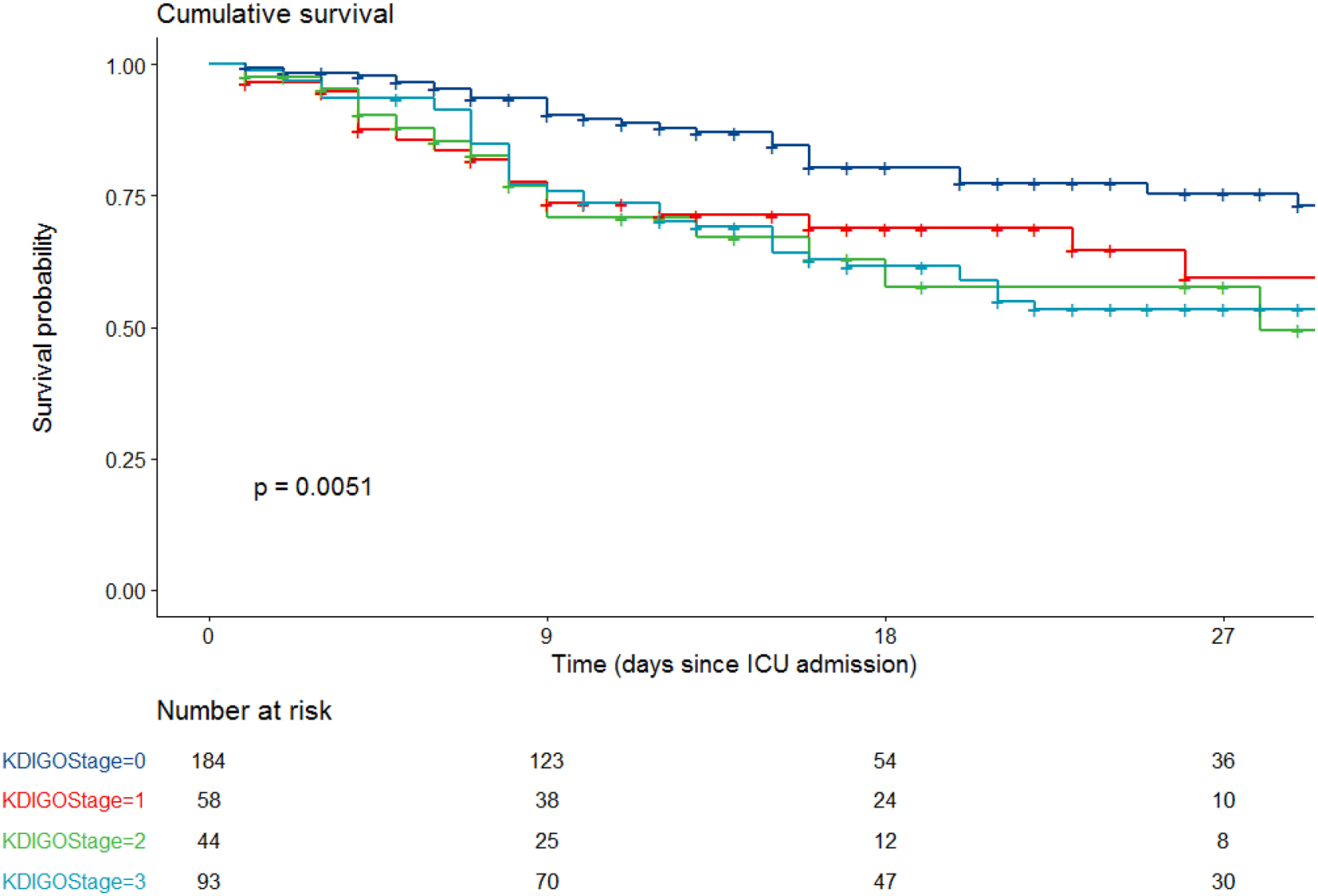
Univariate Kaplan–Meier curves according to KDIGO stages of acute kidney injury

Day 28 mortality in the cohort was 26.4% and was higher in patients with AKI (37.4 vs. 14.7%, *P* < 0.001, Table 2, Fig. 2). Day 28 mortality in patients with AKI stage 1, 2, and 3 was of, respectively, 31% (*n* = 18/58), 34.1% (*n* = 15/44) and 43% (*n* = 40/93) (*p* = 0.005) (Fig. 3 and Additional file 1: Figure S1).

After adjustment for confounders, neither AKI (HR 1.35; 95% CI 0.78–2.32; Table 3) nor AKI stage were associated with mortality (HR [95% CI] for stage 1, 2 and 3 when compared to no AKI of, respectively, 1.02 [0.49–2.10], 1.73 [0.81–3.68] and 1.42 [0.78–2.58]).

**Table 3:** Factors associated with day 28 mortality

Variable	HR	95% CI	*P* Value
Age per year	1.05	1.02–1.07	< 0.001
Normal troponin at ICU admission	0.42	0.25–0.70	0.001
COPD	2.67	1.37–5.22	0.004
Underlying immune defect	1.97	1.22–3.18	0.005
AKI	1.35	0.78–2.32	0.28
Delay since disease onset < 8 days	Ref	–	NA
Delay since disease onset 8 to 14 days	0.51	0.35–0.80	0.004
Delay since disease onset > 14 days	0.39	0.15–0.98	0.05
Center effect (frailty)			0.02

Center was entered as frailty effect

As sensitivity analysis, when Chronic Kidney Disease was forced in the final model, this later was not retained (HR 1.48; 95% CI 0.85–2.58), did not change the final model or influence of AKI on mortality and no interaction between AKI and CKD was observed.

## Discussion

In the present study including 379 ICU patients with SARS-CoV2 pneumonia-related acute respiratory failure, AKI occurred in 52% of cases and was severe in most cases. While chronic kidney diseases and organ failure support were independently associated with AKI occurrence, AKI was not associated with day 28 survival in multivariable analysis.

### Incidence of AKI

AKI is a frequent complication occurring in COVID-19 patients. While initial reports indicated that kidney involvement was negligible [1, 14], it now appears to account for 20 up to 40% of patients [15–17] admitted to the hospital. In the present study, we observed a higher incidence of AKI almost due to the fact that we only included patients admitted to the ICU for severe respiratory failure. In an international case series including patients admitted to the ICU, Chaïbi et al. observed similar incidence as 105/211 (49.8%) patients suffered AKI within the first 7 days after ICU admission; among those, half of them (*n* = 55) had KDIGO3 AKI [18]. This proportion has been evaluated up to 76% of patients admitted to the ICU in New York City [19].

Besides specific glomerular involvement [5, 6] relying to collapsing glomerulopathy related to cytopathogenic effect of SARS-CoV2 on podocytes [20], tubular injury remains the leading cause of AKI in this setting [7]. Such injuries are related to multiple factors as acute right ventricular failure, hypovolemia, proinflammatory cytokines release, endothelial dysfunction, hypercoagulability and mechanical ventilation settings [21].

### Factors associated with AKI

In the present study, we assessed factors associated with AKI occurrence. We observed that life-sustaining therapies as vasopressors and mechanical ventilation as well as preexisting chronic kidney disease were independently associated with AKI. Interestingly, these results did not differ from what we already know in other ICU patients. This may be an informative illustration of the concept of lung–kidney interaction, relying on the direct consequences of hypoxemia, lack of decarboxylation, inflammatory cytokines release—biotrauma—as well as the direct effect of mechanic ventilation settings on renal perfusion, especially in a context, where acute right ventricular failure is frequent [22, 23]. The latter is all the more interesting that we observed that a normal blood troponin level at ICU admission was negatively associated with AKI occurrence. This would reinforce, if we consider an increased troponin level associated with cardiac involvement of SARS-CoV2 infection, the deleterious effect of venous congestion, especially in mechanically ventilated patients. It is to note that we were not able to provide a quantitative relationship between blood level of troponin and AKI that would have reinforced our hypothesis. This is especially related to the multicenter design of the study and the differences in troponin assays used in the different centers. Last, we observed that the longer the delay since disease onset, the lower the probability to suffer from AKI. This might be related to the severity of the respiratory status as we already observed that patients with severe SARS-CoV2 infection admitted within 7 days of disease onset had a higher mortality rate [8].

### AKI and outcome in COVID-19 patients

In the present study, we observed no association between AKI and day 28 outcome. This result is quite discordant with the previously reported ones. Cheng et al. have reported an association between AKI and mortality in a severity-dependent manner [17]. Several other papers reported similar results [13, 19]. Such a lack of association should raise several hypothesis. First, we should acknowledge we could have missed a real relationship we were not able to observe due to the lack of power of our study. We cannot exclude either that the interaction between AKI and the other life sustaining therapies as vasopressors and mechanical ventilation had precluded us to observe a significant relationship. Second, the onset of AKI should be questioned. Indeed, we observed in the present study the lack of association between AKI at ICU admission and outcome. However, it has been already demonstrated that the relative prognostic weight of AKI was predominant when AKI occurred lately during ICU stay [24]. Hirsch et al. reported two third of patients suffered from AKI after the first 24 h and the median time of initiation of dialytic support from hospital admission was 2 [interquartile range -2–141] days {Hirsch:2020ea}. Moreover, most of patients who required mechanical ventilation and developed AKI had the onset of AKI within 24 h of intubation. Taken together, this could plead for an underestimation of the prognostic impact of AKI on outcome in our study.

## Limitations

We acknowledge several limitations. First, we cannot draw any causal conclusion regards to the retrospective design of the study. We adjusted for most of the confounders we collected to better appreciate the relationship between patient and ICU factors and AKI occurrence. Second, neither biomarkers nor markers of glomerula (proteinuria and hematuria) or tubular injury were collected. We would probably have measured a much higher incidence of renal involvement in our cohort. Similarly, we did not provide any pro-inflammatory biomarkers as interleukins 1, 6 or 17. However, we did not observe any independent relationships between daily life inflammation biomarkers and outcome. Third, we did not collect the use of renin–angiotensin–aldosteron system inhibitors. We can assume a large proportion of the patients included in the analysis used to take such medications and we cannot rule out a potential relationship AKI occurrence during ICU stay. Fourth, we did not collect longitudinal data about renal function. While this would have been informative, previously reported data have already suggested that AKI occurred mostly within the first 48 h of ICU stay [25, 26]. Last, we were not able to evaluate the relationship between mechanical ventilation settings and AKI.

## Conclusion

In this large cohort of SARS-CoV2-related pneumonia patients admitted to the ICU, AKI was frequent, mostly driven by preexisting chronic kidney disease and life sustaining therapies, with unclear adjusted relationship with day 28 outcome.

## Supplementary Information

**Additional file 1: Figure S1.** Flow chart of the study. **Figure S2.** Calibration of the mixed-effects multivariable model. Calibration plots shows the predicted probability of the outcome calculated from the multivariable model against the events density.

## Data Availability

The data sets generated and analysed during the current study are not publicly available.

## Abbreviations

ICU: Intensive Care Unit
AKI: Acute kidney injury
