# Supplementary figures and images for: Acute kidney injury in SARS-CoV2-related pneumonia ICU patients: a retrospective multicenter study
Source: Ann Intensive Care. 2021 May 31;11:86. doi: 10.1186/s13613-021-00875-9 (PMC8165682; doi:10.1186/s13613-021-00875-9)

## Slide 1
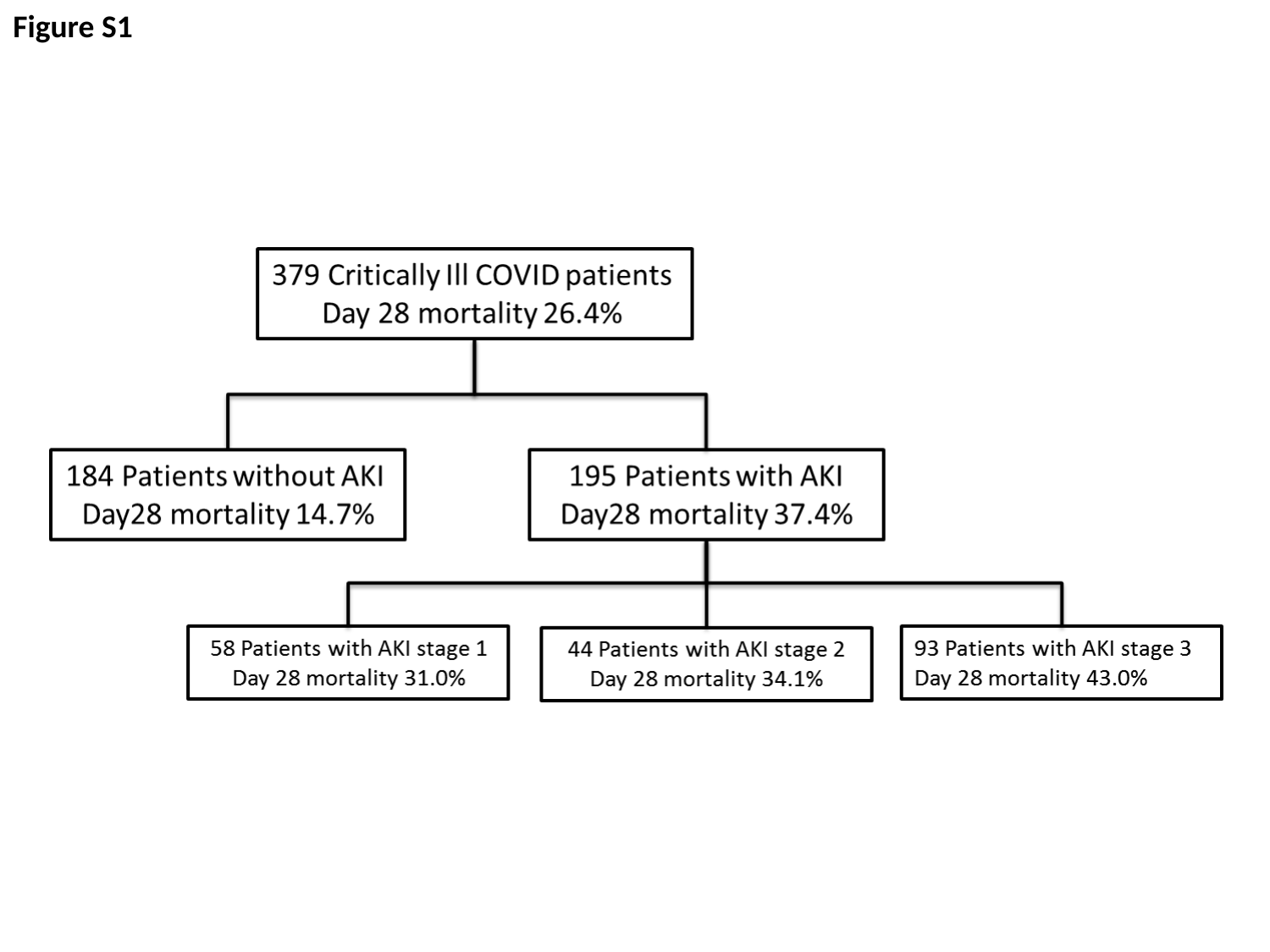

Figure S1

## Slide 2
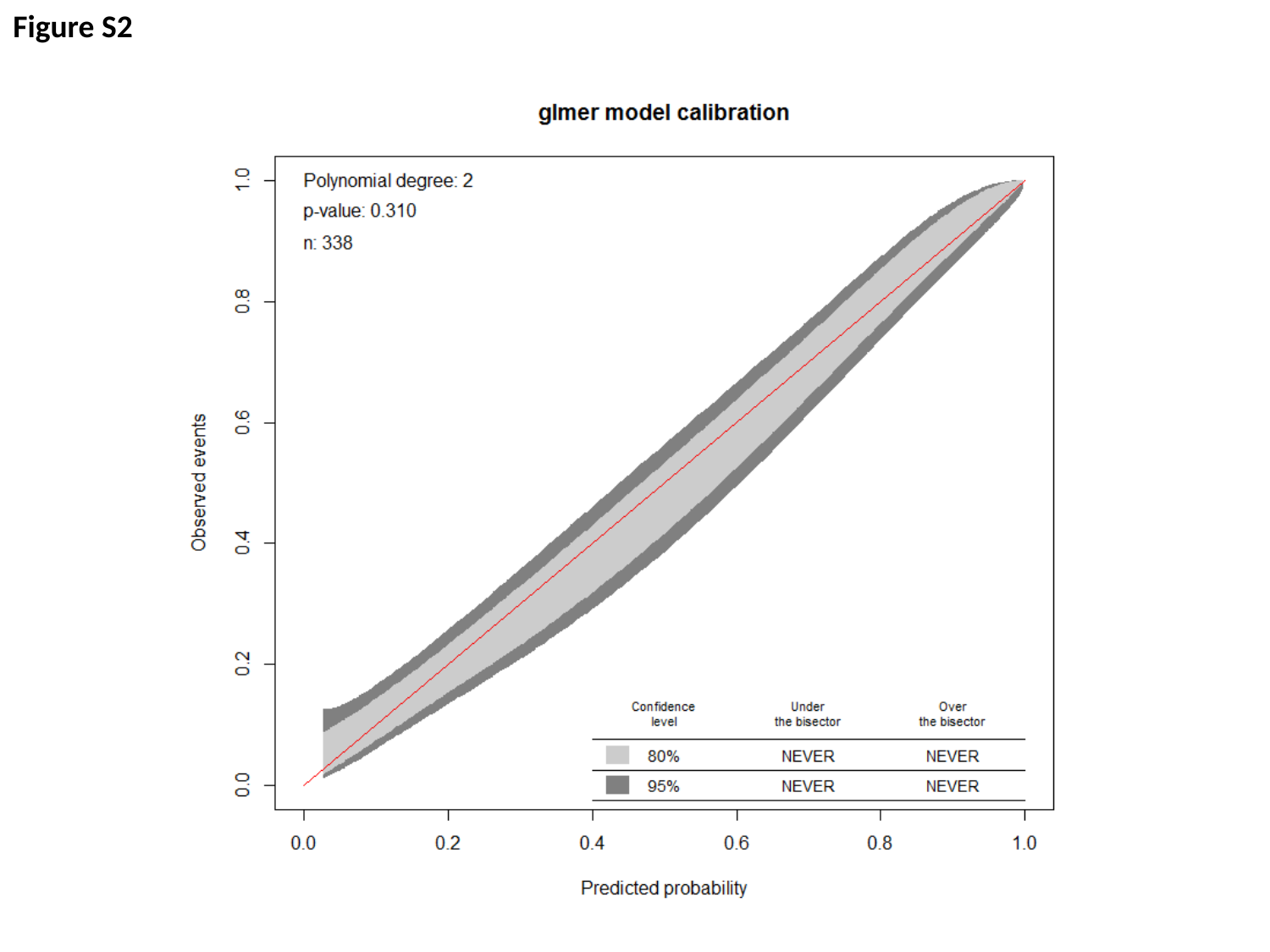

Figure S2

Supplement: Supplementary file 1 — Additional file 1: Figure S1. Flow chart of the study. Figure S2. Calibration of the mixed-effects multivariable model. Calibration plots shows the predicted probability of the outcome calculated from the multivariable model against the events density. [file 13613_2021_875_MOESM1_ESM.pptx]
